# Toward Using Twitter for Tracking COVID-19: A Natural Language Processing Pipeline and Exploratory Data Set

**DOI:** 10.2196/25314

**Published:** 2021-01-22

**Authors:** Ari Z Klein, Arjun Magge, Karen O'Connor, Jesus Ivan Flores Amaro, Davy Weissenbacher, Graciela Gonzalez Hernandez

**Affiliations:** 1 Department of Biostatistics, Epidemiology, and Informatics Perelman School of Medicine University of Pennsylvania Philadelphia, PA United States

**Keywords:** natural language processing, social media, data mining, COVID-19, coronavirus, pandemics, epidemiology, infodemiology

## Abstract

**Background:**

In the United States, the rapidly evolving COVID-19 outbreak, the shortage of available testing, and the delay of test results present challenges for actively monitoring its spread based on testing alone.

**Objective:**

The objective of this study was to develop, evaluate, and deploy an automatic natural language processing pipeline to collect user-generated Twitter data as a complementary resource for identifying potential cases of COVID-19 in the United States that are not based on testing and, thus, may not have been reported to the Centers for Disease Control and Prevention.

**Methods:**

Beginning January 23, 2020, we collected English tweets from the Twitter Streaming application programming interface that mention keywords related to COVID-19. We applied handwritten regular expressions to identify tweets indicating that the user potentially has been exposed to COVID-19. We automatically filtered out “reported speech” (eg, quotations, news headlines) from the tweets that matched the regular expressions, and two annotators annotated a random sample of 8976 tweets that are geo-tagged or have profile location metadata, distinguishing tweets that self-report potential cases of COVID-19 from those that do not. We used the annotated tweets to train and evaluate deep neural network classifiers based on bidirectional encoder representations from transformers (BERT). Finally, we deployed the automatic pipeline on more than 85 million unlabeled tweets that were continuously collected between March 1 and August 21, 2020.

**Results:**

Interannotator agreement, based on dual annotations for 3644 (41%) of the 8976 tweets, was 0.77 (Cohen κ). A deep neural network classifier, based on a BERT model that was pretrained on tweets related to COVID-19, achieved an F_1_-score of 0.76 (precision=0.76, recall=0.76) for detecting tweets that self-report potential cases of COVID-19. Upon deploying our automatic pipeline, we identified 13,714 tweets that self-report potential cases of COVID-19 and have US state–level geolocations.

**Conclusions:**

We have made the 13,714 tweets identified in this study, along with each tweet’s time stamp and US state–level geolocation, publicly available to download. This data set presents the opportunity for future work to assess the utility of Twitter data as a complementary resource for tracking the spread of COVID-19.

## Introduction

In the United States, the rapidly evolving COVID-19 outbreak, the shortage of available testing, and the delay of test results have presented challenges for actively monitoring the spread of COVID-19 based on testing alone. An approach that has emerged for detecting cases without the need for extensive testing relies on voluntary self-reports of symptoms from the general population [1]. Considering that nearly one of every four adults in the United States already uses Twitter, and nearly half of them use it on a daily basis [2], researchers have begun exploring tweets for mentions of COVID-19 symptoms [3-8]. However, considering the incubation period of COVID-19 [9], detecting cases based on symptoms may not maximize the potential of Twitter data for real-time monitoring. The objective of this study was to develop, evaluate, and deploy a natural language processing (NLP) pipeline that automatically collects tweets reporting personal information more broadly—that is, beyond symptoms—that might indicate exposure to COVID-19 in the United States. In this paper, we present a publicly available data set containing 13,714 tweets that were identified by our automatic NLP pipeline between March 1 and August 21, 2020, with each tweet’s time stamp and US state–level geolocation. This data set presents the opportunity to explore the use of Twitter data as a complementary resource “to understand and model the transmission and trajectory of COVID-19” [10].

## Methods

### Data Collection and Annotation

The Institutional Review Board (IRB) of the University of Pennsylvania reviewed this study and deemed it to be exempt human subjects research under Category (4) of Paragraph (b) of the US Code of Federal Regulations Title 45 Section 46.101 for publicly available data sources (45 CFR §46.101(b)(4)).

Between January 23 and March 20, 2020, we collected more than 7 million publicly available tweets that mention keywords related to COVID-19, are posted in English, are not retweets, and are geo-tagged or have user profile location metadata. We developed handwritten regular expressions (Multimedia Appendix 1)—search patterns designed to automatically match text strings—to identify a subset of the 7 million tweets that indicate that the user potentially has been exposed to COVID-19. Our query patterns were designed primarily to help identify potential cases of COVID-19 that are not based on testing and, thus, may not have been reported to the Centers for Disease Control and Prevention (CDC) [11]. The regular expressions matched approximately 160,000 (2%) of the 7 million tweets. Approximately 30,000 (19%) of the 160,000 matching tweets were then automatically removed using a system we developed in recent work [12] for filtering out “reported speech” (eg, quotations, news headlines) from health-related social media data.

In preliminary work [13], two annotators annotated a random sample of 10,000 of the 130,000 filtered tweets, and annotation guidelines (Multimedia Appendix 2) were developed to help the annotators distinguish between three classes of tweets. However, since then, we have removed 1024 of the annotated tweets that were collected from the Twitter Streaming application programming interface (API) based on a keyword that we have stopped using, and we have unified two of the classes. “Potential case” tweets include those that indicate that the user or a member of the user’s household was denied testing for COVID-19, showing symptoms of COVID-19, potentially exposed to presumptive or confirmed cases of COVID-19, or had had experiences that pose a higher risk of exposure to COVID-19. “Other” tweets are related to COVID-19 and may discuss topics such as testing, symptoms, traveling, or social distancing, but do not indicate that the user or a member of the user’s household may be infected. Among the 8976 tweets, 3644 (41%) were annotated by both annotators. Upon resolving the annotators’ disagreements, 1456 (16%) of the tweets were annotated as “potential case” and 7520 (84%) as “other.” Textbox 1 presents (slightly modified) sample tweets that match our handwritten regular expressions and were manually annotated as “potential case.”

**Sample (slightly modified) tweets that match our handwritten regular expressions and were manually annotated as potential cases of COVID-19.**

1. Nearly two weeks ago I had a fever, sore throat, runny nose, and cough. I want to know if it was coronavirus or just the common cold
2. My coworker in next office probably has #coronavirus. He and his wife have the symptoms, but they went to the hospital to get tested and were refused.
3. This girl in my class had the coronavirus, so I’m making an appointment with my doctor for a check up
4. Pretty sure I had a patient tonight with Coronavirus. Had all the symptoms and tested negative for the flu.
5. Why can celebrities, sports athletes & politicians without symptoms get tested, but my symptomatic child who has a compromised immune system cannot? #coronavirus
6. Since getting back from Seattle I’ve been sick and want to get a #coronavirus check. Called my PCP, they said to call health dept. Called them, they said I need to go thru my PCP. Called my PCP again, they said they can’t help me
7. I’m convinced I have coronavirus. I’ve been to NYC, Phoenix, and San Diego in the last few weeks. I have a cough, a runny nose, and I’m really hot #covid19
8. Scared of the coronavirus because I have a sore throat and a headache I think its just a cold but I take the tube 4 times a day
9. Can’t even get testing SCHEDULED while self-quarantined (my decision) and having coronavirus symptoms I take train thru New Rochelle to Manhattan
10. I have a bad cold. I went to the doctor, got some medications, the norm. But they couldn’t rule out coronavirus because they don’t have the tests.

As Textbox 1 illustrates, our handwritten regular expressions are based on query patterns designed to identify tweets that report personal information that may be useful for tracking potential cases of COVID-19, including not only symptoms (tweet 1), but also exposure to potential cases and a lack of access to COVID-19 testing. For example, our regular expressions retrieve tweets reporting that the user may have come in contact with coworkers (tweet 2), classmates (tweet 3), patients (tweet 4), and family members (tweet 5) who may have COVID-19, and potential exposure to COVID-19 through traveling (tweets 6 and 7) and commutes (tweets 8 and 9). Our regular expressions also retrieve tweets reporting that the user (tweet 9 and 10), a family member (tweet 5), or someone else that the user has been in contact with (tweet 2) was denied access to testing, even though they are sick. Since none of the tweets in Textbox 1 report being tested for or diagnosed with COVID-19, they represent potential cases that may not have been reported to the CDC.

### Automatic Classification and Geolocation

**Figure 1:**
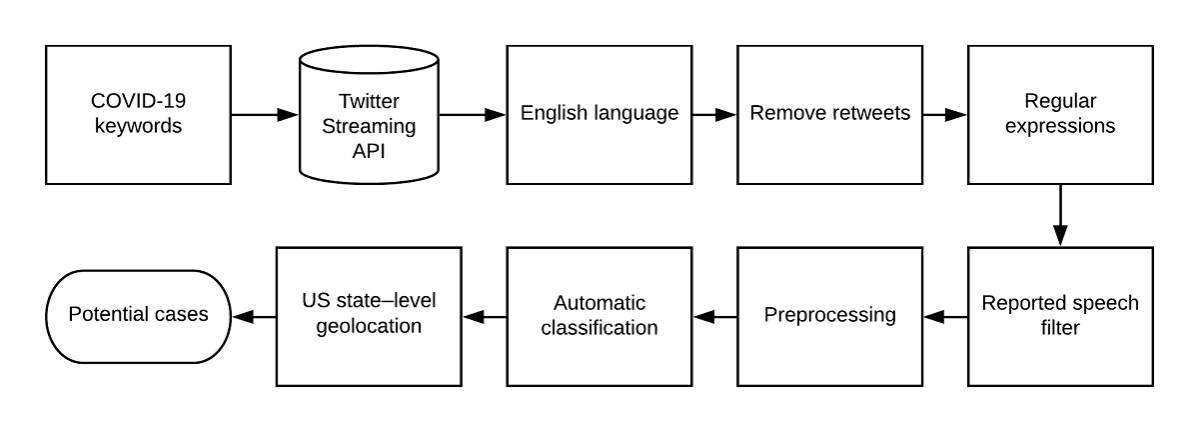
Automatic natural language processing (NLP) pipeline for detecting tweets that self-report potential cases of COVID-19 in the United States.

We split the 8976 annotated tweets into 80% (7181 tweets) and 20% (1795 tweets) random sets—a training set (Multimedia Appendix 3) and held-out test set, respectively—for automatic classification. We used the *ktrain* [14] Python library to train and evaluate two supervised deep neural network classifiers based on bidirectional encoder representations from transformers (BERT): BERT-Base-Uncased [15] and COVID-Twitter-BERT [16]. After feeding the sequence of tweet tokens to BERT, the encoded representation is passed to a dropout layer (dropping rate of 0.1), followed by a dense layer with 2 units and a softmax activation, which predicts the class for each tweet. For training, we used Adam optimization with rate decay and warm-up. We used a batch size of 64, training runs for 3 epochs, and a maximum learning rate of 1 × 10^-5^. We fine-tuned all layers of the transformer model with our annotated tweets. Prior to automatic classification, we preprocessed the tweets by normalizing usernames and URLs, and lowercasing the text. Figure 1 illustrates our automatic NLP pipeline for detecting tweets that indicate potential cases of COVID-19 in the United States. We deployed the pipeline on more than 85 million unlabeled tweets that were continuously collected between March 1 and August 21, 2020. We used Carmen [17] to infer the geolocation—at the US state level—of tweets that the classifier predicted as potential cases.

## Results

Interannotator agreement, based on dual annotations for 3644 (41%) of the 8976 tweets, was 0.77 (Cohen κ), considered “substantial agreement” [18]. We evaluated two deep neural network classifiers on a held-out test set of 1795 (20%) of the 8976 tweets. The classifier based on the BERT-Base-Uncased pretrained model achieved an F_1_-score of 0.70 (precision=0.72, recall=0.67) for the “potential case” class, and the classifier based on the COVID-Twitter-BERT pretrained model achieved an F_1_-score of 0.76 (precision=0.76, recall=0.76), where:


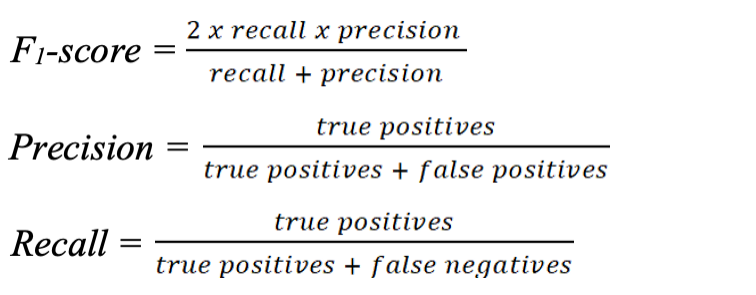


**Figure 2:**
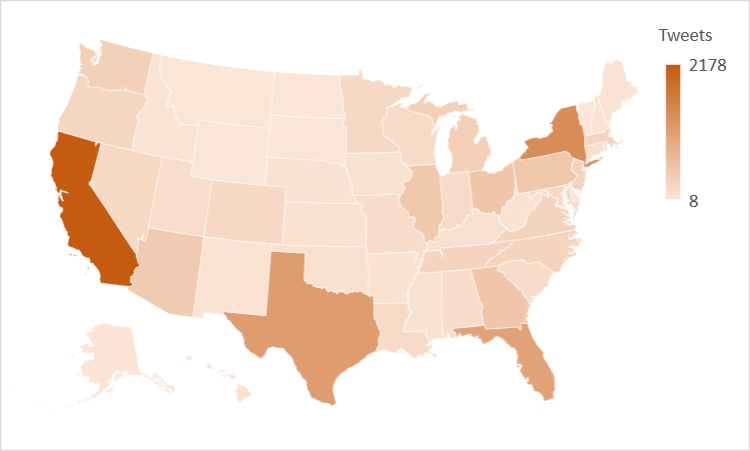
Tweets self-reporting potential cases of COVID-19 in the United States, by state, between March 1 and August 21, 2020.

We deployed our automatic pipeline, using the COVID-Twitter-BERT classifier, on more than 85 million unlabeled tweets that were continuously collected from the Twitter Streaming API between March 1 and August 21, 2020. Among the subset of tweets that were posted in English, not retweets, matched the regular expressions, and were not filtered out as reported speech, the COVID-Twitter-BERT classifier detected 13,714 “potential case” tweets for which Carmen inferred a US state–level geolocation. Figure 2 illustrates the ranges of “potential case” tweets that were automatically detected per state. We automatically detected “potential case” tweets from all 50 states, with the highest numbers posted in California, New York, Texas, and Florida.

## Discussion

### Principal Findings

While Twitter data has been used to identify self-reports of symptoms by people who have tested positive for COVID-19 [3,4], the shortage of available testing and the delay of test results in the United States motivated us to assess whether Twitter data could be scaled to identify potential cases of COVID-19 that are not based on testing and, thus, may not have been reported to the CDC. There are studies that have not limited their exploration of COVID-19 symptoms on Twitter to users who have tested positive for COVID-19 [5-8]; however, limiting the detection of potential cases to symptoms may still underutilize the information available on Twitter. Our automatic NLP pipeline has detected potential cases of COVID-19 across the entire United States that are neither based on testing nor limited to symptoms, providing the opportunity to explore the utility of Twitter data more broadly as a complementary resource for tracking the spread of COVID-19. An analysis based on this data set is beyond the scope of this study. The 13,714 “potential case” tweets identified in this study can be downloaded using a Python script [19] and the input file in Multimedia Appendix 4, which contains the user ID, tweet ID, time stamp, and inferred state-level geolocation for each tweet. The script downloads the tweets that are still publicly available.

### Conclusions

This paper presented an automatic NLP pipeline that was used to identify 13,714 tweets self-reporting potential cases of COVID-19 in the United States between March 1 and August 21, 2020, that may not have been reported to the CDC. This publicly available data set presents the opportunity for future work to assess the utility of Twitter data as a complementary resource for tracking the spread of COVID-19.

## Appendix

Multimedia Appendix 1 Regular expressions.

Multimedia Appendix 2 Annotation guidelines.

Multimedia Appendix 3 Training data.

Multimedia Appendix 4 Exploratory Twitter data set.

## Abbreviations

API: application programming interface
BERT: bidirectional encoder representations from transformers
CDC: Centers for Disease Control and Prevention
NLP: natural language processing
